# High-Frequency Stimulation of the Ventral Tegmental Area Rescues Respiratory Failure

**DOI:** 10.21203/rs.3.rs-6740056/v1

**Published:** 2025-06-23

**Authors:** Hojin Shin, Sara vettleson-trutza, Juan Rojas Cabrera, Youngjong Kwak, kristen Scheitler, Sheng-Ta Tsai, Tyler Oesterle, Jaeyun Sung, Charles Blaha, Yoonbae Oh, Kendall Lee

**Affiliations:** Mayo Clinic; Mayo Clinic; Mayo Clinic; Mayo Clinic; Mayo Clinic; Mayo Clinic; Mayo Clinic; Mayo Clinic; Mayo Clinic; Mayo Clinic in Rochester; Mayo Clinic

## Abstract

**Introduction:**

Opioid use disorder constitutes a significant health crisis in the United States, contributing to high rates of opioid overdose-related deaths. A major driver of these fatalities is fentanyl, a potent synthetic opioid with both sedative and analgesic properties. These properties that make fentanyl clinically effective also increase its addictive potential. Early in opioid addiction, abnormal increases in extracellular dopamine in the nucleus accumbens (NAc) reinforce excessive drug-seeking behaviors which can lead to fatal respiratory depression. Given this mechanism, we investigated whether high frequency stimulation (HFS), mimicking human deep brain stimulation (DBS) of the ventral tegmental area (VTA) could block NAc dopamine increase following an acute lethal dose of fentanyl. We hypothesized that VTA DBS would mitigate these dopaminergic responses and prevent fentanyl-induced respiratory failure.

**Methods:**

Multiple cyclic square wave voltammetry (M-CSWV), was applied via a carbon fiber microelectrode in the NAc of urethane-anesthetized male Sprague-Dawley rats. Dopamine levels were recorded at baseline and following acute fentanyl (30 μg/kg, i.v.). HFS (130 Hz frequency, 200 μsec pulse width, and 0.2 mA amplitude) was administered to the VTA before and during fentanyl exposure.

**Results:**

Acute fentanyl administration resulted in a 178.2% increase in NAc dopamine levels from baseline, accompanied by a decline in respiratory rates to critically low levels (45 breaths per minute vs. 102 bpm), eventually resulting in 100% mortality. HFS of the VTA did not significantly alter baseline tonic dopamine levels or prevent fentanyl-induced dopamine increase in the NAc but was able to fully rescue fentanyl-induced respiratory failure.

## Introduction

Opioid use disorder (OUD) is a major public health crisis in the United States (US) ^1^, ^2^. In 2017, $1 trillion of the U.S. economy was allocated towards addressing opioid-related comorbidities, treatments, and deaths ^1^. Fentanyl, a synthetic opioid, is commonly prescribed for pain management in cancer patients and used for surgical procedures due to its potent activation of mu-opioid receptors (MORs) ^3^. The high lipophilicity of fentanyl allows it to rapidly cross the blood-brain barrier, causing nearly immediate pain relief ^4^. Notably, fentanyl, is approximately 50–100 times more potent than oxycodone ^5^, which significantly increases its risk of addiction and overdose.

MORs are located throughout the brain, including regions like the nucleus accumbens (NAc) and ventral tegmental area (VTA), which are a part of the brain’s mesolimbic reward system and have been heavily implicated in addiction^6^. Previous studies have demonstrated that fentanyl modulates accumbal dopamine via its inhibition of GABAergic interneurons in the VTA, leading to the disinhibition of dopaminergic projections synapsing onto neurons in the NAc^7^,^8^. This increase in dopamine release within the NAc is closely linked to the positively reinforcing effects of fentanyl^8^. Researchers have also suggested that the association between pain relief and fentanyl use may also help to behaviorally reinforce the cycle of addiction through Pavlovian conditioning^9^. In this process, fentanyl not only triggers the release of neurotransmitters like dopamine, which is linked to reward and drug-seeking behaviors, but also becomes strongly associated with pain relief mediated by the mu-1 receptor subtype, further driving its compulsive use, which is the hallmark of addiction^10^. Unfortunately, when activated by potent opioids like fentanyl, MORs can also inhibit respiratory function mediated by the mu-2 receptor subtype, which significantly contributes to opioid-associated mortality^11^. Indeed, fentanyl alone accounts for over 70% of drug-related overdose deaths in North America^12^.

One promising avenue for treatment-resistant OUD (TROUD) intervention is deep brain stimulation (DBS), a neuromodulatory technique involving the targeted implantation of electrodes to deliver controlled electrical stimulation to deep brain structures. Patients with TROUD do not respond to standard medication assisted therapies and psychosocial interventions, warranting the investigation of novel treatment approaches^13^. DBS has been successful in treating movement disorders such as Parkinson’s disease and essential tremor, as well as psychiatric conditions including obsessive-compulsive disorder, Tourette’s syndrome, and more recently, addiction^14–16^. A clinical trial by Rezai et al. demonstrated the potential of DBS in OUD by targeting the NAc in treatment-resistant patients^17^, yielding craving reduction and prolonged abstinence. However, unlike DBS applications for movement disorders symptomatic relief in addiction is less objective and challenging to monitor due to lack of internal biomarkers and an unclear therapeutic mechanism^18^. Neurochemicals such as dopamine, GABA, glutamate, and serotonin, along with electrophysiological neuronal activity, have been proposed as potential biomarkers for monitoring the effects of DBS on the brain^19^. Previous studies have demonstrated the importance of both phasic and extracellular (tonic) dopamine levels in driving reward and sustained motivation^20^. Accurately monitoring dopamine levels with high spatiotemporal resolution over extended periods of time is essential for understanding decision-making during addiction-related behaviors^21^,^22^. However, no traditional voltammetric methods have been capable of directly measuring tonic neurochemical levels in near real-time until now.

Multiple cyclic square wave voltammetry (M-CSWV), is an electrochemical voltammetric sampling technique for measuring tonic dopamine levels and, in combination with recording microelectrodes, offers higher spatiotemporal resolution with less tissue trauma than traditional methods like microdialysis^23^. Building on prior work demonstrating that VTA DBS modulates oxycodone-induced dopamine levels and respiration^24^, we hypothesize that VTA DBS would similarly modulate fentanyl-induced increases in tonic extracellular dopamine levels and prevent respiratory depression. This study aims to elucidate the effects of DBS in the VTA on dopamine dynamics and respiratory responses following acute fentanyl exposure.

## Materials and Methods

### Subjects

Adult male Sprague–Dawley rats (n = 12) weighing 220–400g (Envigo, IN, USA) were utilized in this study. Rats were group-housed by sex in an Association for Assessment and Accreditation of Laboratory Animal Care International (AAALAC)-accredited vivarium. They were maintained on a standard 12-hour light/dark cycle at constant temperature (21°C) and humidity (45%), with ad libitum access to food, water, and enrichment materials (wood chews, bedding, yogurt, and fruit gem treats). Animal studies were approved by the Institutional Animal Care and Use Committee (IACUC) at Mayo Clinic, Rochester, and adhered to the NIH Guide for the Care and Use of Laboratory Animals guidelines (Department of Health and Human Services, NIH publication No. 86 – 23, revised 1985).

### Dopamine sensing microelectrode fabrication

Carbon fiber microelectrodes (CFMs) were fabricated following previously established design protocols at Mayo Clinic^23^,^25^. CFMs were stabilized and pretested in a sodium chloride buffer prior to coating with a PEDOT:Nafion deposition solution^27^ to minimize biofouling in vivo.

### Implantation of recording and stimulating electrode

Rats were anesthetized with an intraperitoneal dose of urethane (1.5 g/kg^26,28^; Sigma-Aldrich, St Louis, MO, USA). Ophthalmic ointment was applied to both eyes to prevent corneal desiccation. Hind-paw and tail pinch were used to confirm the surgical plane of anesthesia. Animals were then positioned in a stereotaxic frame (David Kopf Instruments, Tujunga, CA, USA). Respiratory rate was continuously monitored using RespiRAT^24^ (Intuitive Measurement Systems, AZ, USA) to monitor the physiological state and depth of anesthesia throughout the experiment. Artificial ventilation was not used for any of the experiments. Three burr holes were drilled based on a standard rat brain atlas^29^: the first for placement of a CFM into the NAc (all coordinates from bregma: AP 1.2 mm, ML 2.0 mm, DV 6.5–7.5 mm from dura), the second for insertion of a concentric stimulating electrode (SS-SNE-100, Microprobes for Life Sciences, USA) into the VTA (AP −5.3 mm, ML 0.9 mm, DV 7.5–9 mm from skull), and the third for placement of an Ag/AgCl reference electrode in the contralateral cortex (DV: −1 to −2 mm from dura)^30^.

### Recordings and stimulation parameters

The stimulating electrode in the VTA and CFM in the NAc were gradually advanced to confirm correct targeting, and depth was optimized through robust stimulation-evoked dopamine signals measured by fast-scan cyclic voltammetry (FSCV; −0.4 to 1.3 V sweep, 10 Hz, 400 V/s scan rate) using an in-house electrometer and stimulator, MAVEN. High frequency stimulation parameters were biphasic pulses (60 Hz, 2 ms pulse width, 0.2 mA amplitude, and 2 s duration). Once the optimal electrode depths were identified, MAVEN was switched to the M-CSWV sensing technique^23^. After 60 minutes of M-CSWV stabilization, the rats underwent the following experimental conditions.

To test the hypothesis that fentanyl increases tonic dopamine levels in the NAc we injected fentanyl (30 μg/kg, National Institute on Drug Abuse) intravenously (i.v.) over 10–15 sec via a cannula inserted into the tail vein (n = 6). Rats were observed for 30 minutes post-administration. In preclinical studies, fentanyl is used at a wide range of doses and routes of administration. We chose this dose and route of administration previously demonstrated to induce significant respiratory depression and near 100% eventual mortality when rats are not artificially ventilated^31^. It should be noted that rats used in our study were anesthetized with urethane (a general anaesthetic known to modulate respiration in rats^32^) before receiving an acute bolus of fentanyl.

Next, we wanted to test the hypothesis that continuous VTA stimulation (biphasic pulses at 130 Hz, 0.2 ms pulse width, 0.2 mA amplitude) for 30 minutes prior to fentanyl administration would mitigate fentanyl induced increases in tonic dopamine levels in the NAc and prevent respiratory depression (n = 6). The stimulation delivered was interleaved with the M-CSWV recording to minimize artifacts. After 30 minutes of stimulation the M-CSWV waveform signal was restabilized to a new baseline and fentanyl (30 μg/kg i.v.) was administered (as per methods above), and stimulation was continued for another 30 minutes. Subsequently, stimulation was discontinued, and rats were monitored for an additional 30 minutes before euthanasia with pentobarbital (Fatalplus, 390 mg/mL i.v.; 1 mL). Saline controls during stimulation and fentanyl administration demonstrated no significant effects on tonic dopamine levels in our previous study^24^, thus omitted here to minimize experimental duration.

### Calibration of carbon fiber microelectrodes (CFMs)

After completion of each experiment, dopamine fluctuations recorded from CFMs were calibrated in vitro using dopamine solutions of known concentrations (100, 200, and 300 nm), as previously described^23^.

### Histology

Upon experiment completion, the brain was extracted and placed in 4% paraformaldehyde for 1–3 weeks and then placed in a 25% glycerol sinking solution for 24 hours prior to sectioning. Coronal sections were sliced to 40μm using a microtome (Leica Biosystems, U.S.). Sections were then mounted on glass slides, stained with cresyl violet (Sigma-Aldrich, U.S.), and examined with light microscopy to confirm electrode placement post experimentation.

### Statistical analysis

Baseline dopamine levels were determined by averaging the most stable baseline period, defined as a period where variance was within ± 5% (30 M-CSWV measurements). This was used to normalize raw data and calculate average percent change from baseline.

To assess a high dose of fentanyl effects on tonic dopamine levels in the NAc, 6 normalized M-CSWV measurements collected at a stable period prior to fentanyl administration (“Baseline”) were compared to 6 measurements centered around peak dopamine levels after fentanyl administration (“Peak-fentanyl”). Rats expired within 4–5 minutes after fentanyl administration (equivalent to a period of 24 to 30 M-CSWV measurements). Thus, for consistency across animals, comparisons included a one-minute interval around peak dopamine levels (i.e., 3 measurements before the peak, the peak measurements, and 2 measurements after).

We hypothesized that HFS of the VTA would rescue fentanyl induced respiratory depression, allowing for more extensive neurochemical and respiratory data collection. The baseline prior to HFS and fentanyl (Baseline) was the average of 30 minutes or 180 M-CSWV measurements. Dopamine levels during VTA stimulation (HFS on) were determined by averaging 180 measurements prior to fentanyl administration. For direct comparison to fentanyl with no stimulation, 6 measurements post-fentanyl during HFS (HFS on + 30 μg FENT) were averaged. Additionally, 180 measurements after HFS cessation (HFS off) were averaged to assess post-intervention dopamine changes.

Tonic dopamine time-tracing included all data collected and were normalized as described above, that is by averaging dopamine levels for each animal to a stable baseline. Dopamine concentration changes (Δ) were computed as the difference between average values from 6 pre-intervention and 6 post-intervention data points. Respiratory rates were measured by calculating the running average breath rate per minute, averaging every 60 seconds and using 3 consecutive averaged data points for statistical comparisons.

Tonic M-CSWV raw data and color plots were analyzed using MATLAB (MathWorks, Natick, MA, USA). Respiration rate data were extracted from the RespiRat software and analyzed in Microsoft Excel. Figures and statistical analyses were conducted using Prism 10 (GraphPad Software, San Diego, CA, USA) and BioRender.com, and then compiled in Microsoft PowerPoint. Statistical significance was set at P < 0.05 using paired t-tests to compare dopamine and respiratory rate changes before and after fentanyl alone, HFS preceding fentanyl administration, and fentanyl in the presence of HFS. All mean values are presented as mean ± standard error of mean.

## Results

### Cresyl violet stains show accurate final electrode placement in NAc and VTA

**Figure 1:**
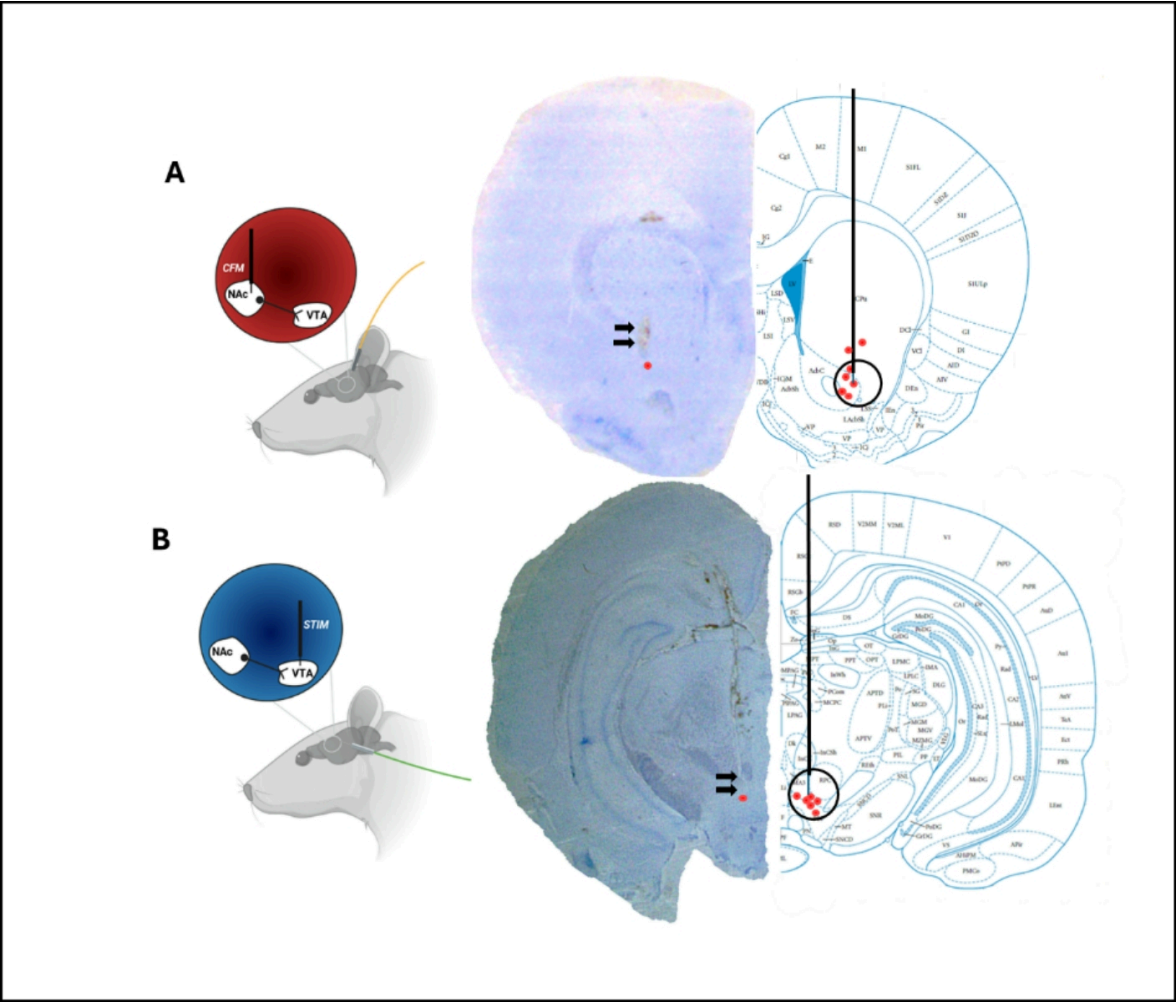
Verification of carbon fiber microelectrode (CFM) and stimulation electrode placements in the nucleus accumbens (NAc) and ventral tegmental area (VTA), respectively A. Cresyl violet staining showing placement of a carbon fiber microelectrode in the nucleus accumbens core, n=7 (labeled AcbC. Red dots on the histological image (right) indicate verified electrode locations overlaid on a coronal brain atlas40. B. Cresyl violet staining showing placement of a stimulating electrode in the ventral tegmental area, n=6 (labeled PBP). Red dots on the histological image (right) indicate confirmed stimulation sites across animals mapped onto the coronal atlas. Black circles highlight electrode locations. The single red point on the histological image (left) represents the end of the electrode trajectory.

To confirm the stereotactic placement of our CFM recording and stimulating electrodes we extracted whole brains from rats after experimental conclusion and preserved them in 4% PFA. Cresyl violet staining demonstrated accurate placement of CFM in NAc coronal slices, with red dots (n = 7) indicating the end of the trajectory (Fig. 1A) VTA targeting was also accurate with minor variance, indicated by red dots (n = 6). (Fig. 1B). Some variation in final electrode placement was expected between rats due to differences in individual rats’ weight and age.

### Fentanyl increases extracellular dopamine levels

**Figure 2:**
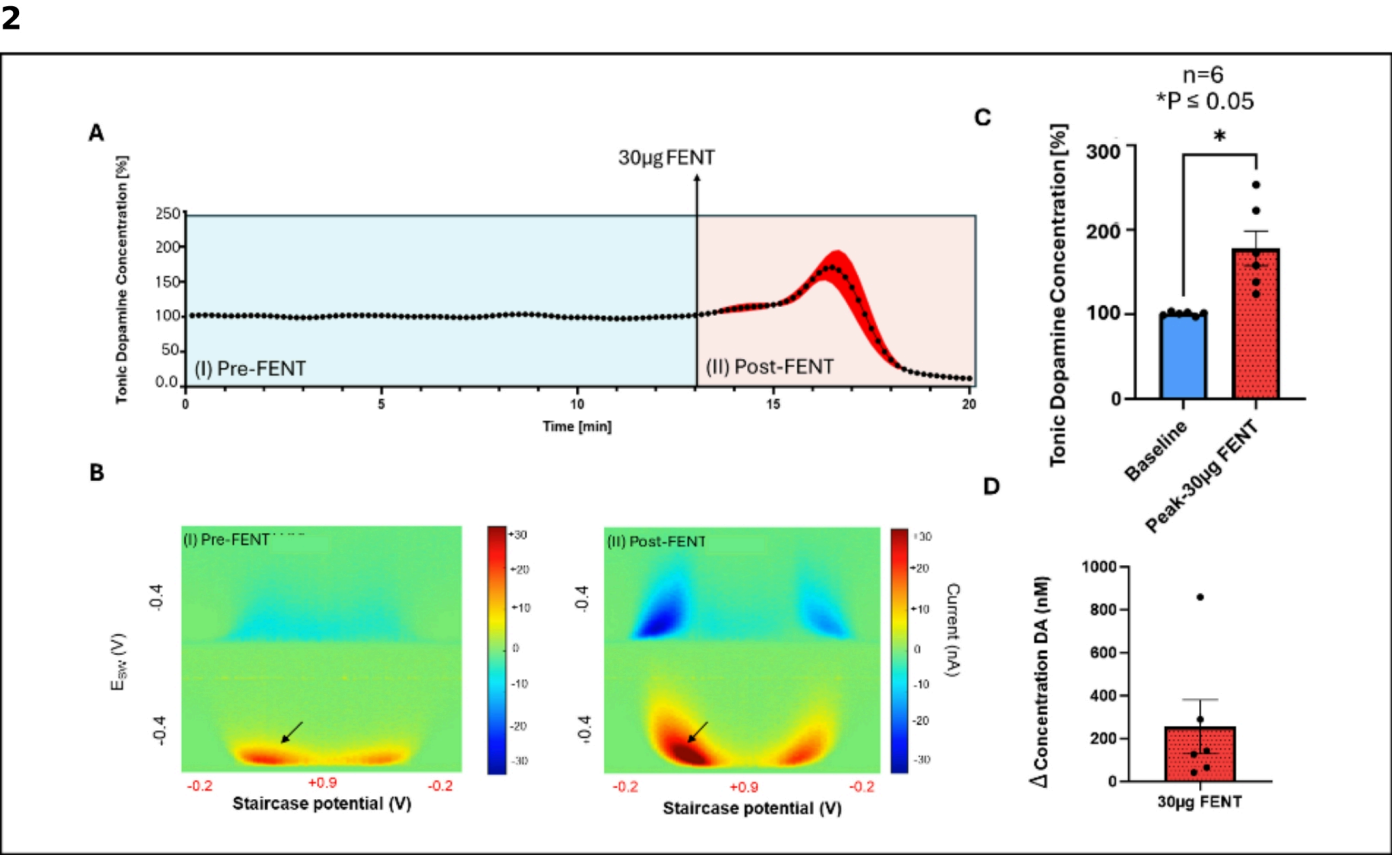
Fentanyl (30 μg i.v.) increases tonic dopamine extracellular levels in the nucleus accumbens (n = 6 rats). A. M-CSWV time trace showing normalized dopamine levels before (I) Pre-fentanyl (baseline) and after (II) Post-fentanyl administration. In all cases, fentanyl administration resulted in 100% mortality. B. Tonic dopamine concentrations normalized from baseline (100%) show a significant peak increase of 178.2% ± 20.4% following 3.5 minutes post fentanyl administration from a baseline 100.8% ± 0.9% C. Representative M-CSWV color plots depicting dopamine oxidation (red) and reduction (blue) before (I) Pre-fentanyl and after (II) Post-fentanyl administration. D. Fentanyl administration evoked a peak change (Δ) in tonic dopamine (DA) concentration of 254 ± 126 nM.

An acute intravenous dose of 30 μg/kg fentanyl was observed to elicit a significant increase in NAc tonic dopamine levels (Fig. 2A). A paired t-test confirmed this increase in dopamine levels from pre-fentanyl (Baseline) to post-fentanyl (Peak-fentanyl) at ~ 3.5minutes after initial administration (P = 0.0132, 178.2% ± 20.4% above baseline, n = 6 rats.) (Fig. 2B). Representative color plots are shown in Fig. 2C highlighting the peak changes, in which red regions indicate higher dopamine concentrations (dopamine oxidation) and blue regions reflect dopamine reduction. The peak change in dopamine concentration is presented in Fig. 2D (Δ = 254 ± 126 nM, n = 6) confirming fentanyl’s potent dopaminergic effects. Fentanyl administration resulted in 100% mortality rate among subjects.

### High-frequency stimulation of the VTA modulates extracellular dopamine levels

Next, we sought to determine if VTA HFS impacts tonic dopamine levels in NAc and if this would attenuate the effects of fentanyl on dopamine levels. Thus, continuous VTA HFS (130 Hz, 0.2 mA) was applied for one hour before administering fentanyl (30 μg/kg i.v.) while tonic dopamine levels in the NAc were recorded by M-CSWV.

**Figure 3:**
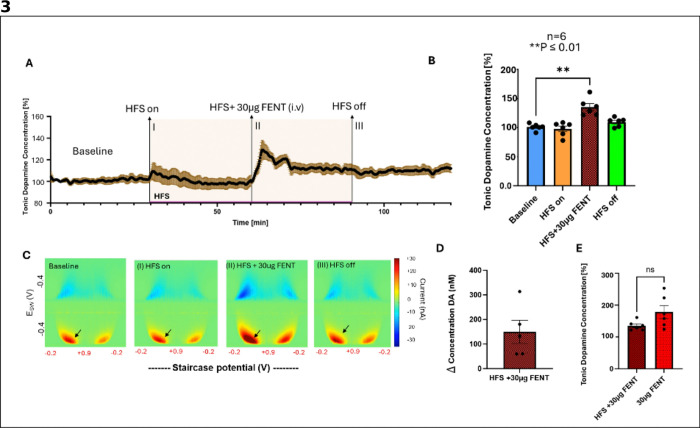
High frequency stimulation (HFS) of the VTA modulates fentanyl-induced tonic extracellular dopamine levels in the nucleus accumbens (n = 6 rats). A. M-CSWV time trace showing normalized dopamine levels before HFS (Baseline), during HFS (I. HFS on), after fentanyl (30 μg i.v.) administration in the presence of HFS (II. HFS + 30 μg FENT), and after cessation of stimulation (II. HFS off). B. Tonic dopamine concentrations normalized from baseline (100%) showing a significant peak increase in dopamine levels following 3.5 minutes post fentanyl administration in the presence of HFS (HFS + 30 μg FENT: 135.2% ± 6%, P = 0.001) and a partial return to baseline after stimulation cessation (HFS off: 109.5% ± 3%, P = 0.052). C. Representative M-CSWV color plots depicting peak changes in dopamine oxidation (red) at baseline, I. HFS on, II. HFS + 30 μg FENT, and III. HFS off. D. Fentanyl administration evoked a peak change (Δ) in dopamine (DA) concentration of 149.5 ± 47 nM E. No significant difference was observed between fentanyl-induced peak changes in dopamine levels with and without VTA HFS (P = 0.0754).

The M-CSWV time trace showed that VTA HFS slightly increased dopamine levels, followed by a gradual decline to just below baseline (Fig. 3A). However, this overall change was not statistically significant (P = 0.4634, n = 6, Fig. 3B). Representative color plots show these variations in dopamine levels at different time points of each experimental condition (Fig. 3C). Following fentanyl administration during HFS (HFS + 30 μg FENT), dopamine levels increased significantly from baseline following ~ 3.5 minutes post fentanyl administration (101.0% ± 2.3–135.2% ± 6% P = 0.001, Fig. 3B). After HFS was discontinued, dopamine levels gradually returned to near baseline, with a slight but non-significant increase remaining (HFS off: 109.5% ± 3%, P = 0.052). The change in dopamine concentration (Δ) is shown in Fig. 3D (149.5 ± 47 nM, n = 5). While HFS appeared to modulate fentanyl-induced dopamine levels, the reduction in dopamine peak levels compared to the fentanyl-only condition was not statistically significant (P = 0.0754, n = 6, Fig. 3E).

### High-frequency stimulation of the VTA rescues fentanyl-induced respiratory failure

**Figure 4:**
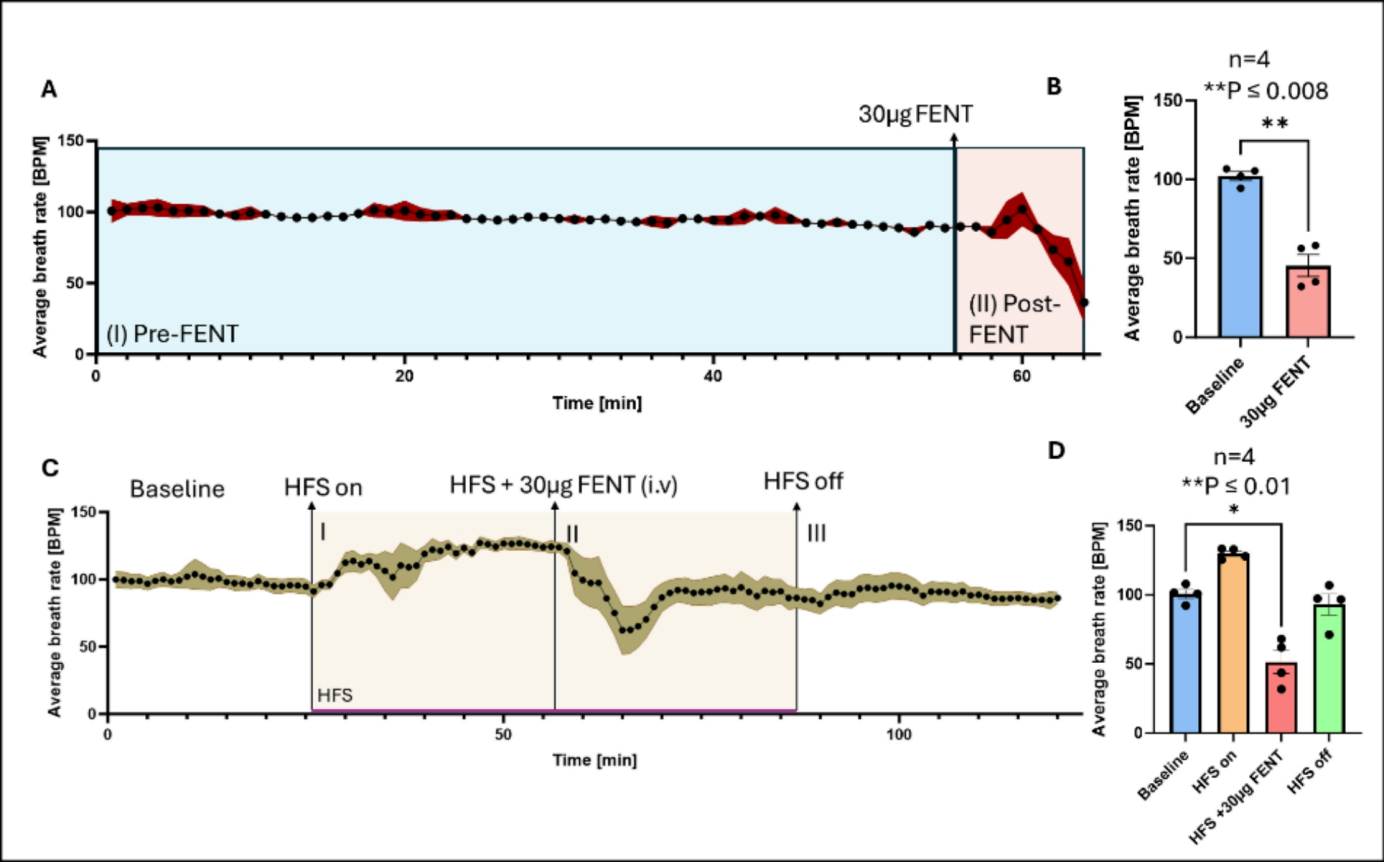
High frequency stimulation (HFS) of the VTA rescues fentanyl (30 μg i.v.) induced respiratory depression (n = 4 rats). A. Time trace of respiratory rate (average breaths per second) before (I) Pre-fentanyl and after (II) Post-fentanyl administration. B. Respiratory rate decline following fentanyl (102.2 ± 2.75 bpm Pre-fentanyl vs. 45.44 ± 6.83 bpm Post-fentanyl, P = 0.0087). C. Time trace of respiratory rate across different experimental stages with HFS, showing a recovery in breathing after fentanyl administration (HFS + 30 μg FENT). D. Respiratory rate comparison across conditions: Compared to baseline (100.6 ± 3.2 bpm), HFS on significantly increased respiratory rate (129.9 ± 1.939 bpm, P = 0.0054). HFS + 30 μg FENT showed a significant decrease (51.42 ± 8.35 bpm, P = 0.0141). Respiration fully recovered to the baseline rate after HFS off (93.04 ± 7.65 bpm), suggesting that HFS rescues fentanyl-induced respiratory failure.

We next examined whether VTA HFS could counteract fentanyl-induced respiratory depression. Respiratory rate was continuously monitored throughout each experiment. In the absence of stimulation, fentanyl (30 μg/kg i.v.) caused a severe respiratory decline (Fig. 4A), reducing average breathing rates from 102.2 ± 2.75 breaths per minute (bpm) to 45.44 ± 6.83 bpm (P = 0.0087, n = 4, Fig. 4B). Without intervention, respiration did not recover, resulting in respiratory failure and 100% mortality. In marked contrast, when VTA HFS was applied before fentanyl administration, respiratory rates stabilized (Fig. 4C) HFS alone significantly increased baseline respiratory rates (129.9 ± 3.2 bpm, P = 0.0054, n = 4, Fig. 4D). When fentanyl was administered in the presence of HFS, initial respiratory depression still occurred reaching a peak decrease in ~ 6 minutes after injection (Fig. 4D), (100.6 ± 3.2 bpm to 51.42 ± 8.35 bpm, P = 0.0141). However, all rats that received HFS survived, and respiration fully recovered to near baseline levels after stimulation ceased (93.04 ± 7.65 bpm, Fig. 4D).

## Discussion

Here, we demonstrate the effects of HFS of the VTA on both fentanyl-induced respiratory depression and extracellular dopamine levels in the NAc in male rats. Unlike our initial hypothesis, HFS of the VTA did not significantly reduce dopamine levels in the NAc after an acute fentanyl injection of 30 μg/kg i.v. After VTA stimulation before any drug administration there was a slight reduction in NAc dopamine levels, but we still recorded large increases after acute fentanyl exposure even with continuous VTA stimulation. Most importantly, HFS of the VTA effectively prevented respiratory failure and mortality at this lethal dose of 30 μg/kg i.v. All rats that received HFS VTA survived, but still experienced significant respiratory depression immediately following fentanyl administration that eventually recovered back to baseline.

### Fentanyl increases extracellular dopamine levels in the NAc

Our results indicate that M-CSWV is able to capture real time changes in extracellular dopamine levels in the NAc in response to a potent opioid like fentanyl. In the presence or absence of HFS, an acute dose of 30 μg/kg i.v. fentanyl significantly increased tonic extracellular levels of dopamine in the NAc (see Figs. 2B, 3B). Fentanyl is a potent MOR agonist^6^. While MORs are distributed throughout the central and peripheral nervous system they are particularly expressed presynaptically on inhibitory GABAergic interneurons in the VTA^33^. Typically, these GABAergic interneurons exert a tonic inhibitory action on VTA dopamine neurons to regulate dopamine levels in the NAc^33^. However, fentanyl binding presynaptically to MORs leads to a strong hyperpolarization of these GABAergic interneurons via inhibition of voltage-gated calcium channels^33^. This reduces GABA release, effectively disinhibiting VTA dopamine neurons. As a result, there is an increase in VTA dopamine neuron firing, leading to an elevation in extracellular dopamine levels in the NAc^33^.

### Potential role of brain hypoxia on extracellular dopamine levels in the NAc

HFS of the VTA failed to significantly attenuate fentanyl-induced increases in dopamine levels in the NAc compared to fentanyl alone. In both stimulation and stimulation absent conditions rat respiratory rate significantly dropped below baseline within 5 minutes after fentanyl injection. The magnitude of this drop was similar to the level of increased NAc dopamine in both stimulation and stimulation absent conditions (see Figs. 2A, 2B, 3A, 3B, and 4A-D) suggesting that these results may reflect hypoxia-induced alterations in dopamine release^34^, which could have masked the effects of fentanyl alone on tonic dopamine levels in the NAc. While M-CSWV is selective for dopamine detection, hypoxic conditions in the brain could trigger an exaggerated dopamine response beyond the direct effects of fentanyl^34^.

A significant drop in respiratory rate can lead to acute hypoxic conditions in the brain ^35^,^36^. Lack of oxygen in the brain may have suppressed the ability of HFS of the VTA to significantly mitigate increases in NAc dopamine after a high dose fentanyl injection. Further dose-response studies would help establish a clearer relationship between fentanyl-induced dopamine level fluctuations and VTA stimulation.

### Stimulation of the VTA modulates extracellular dopamine through heterogenous neurocircuitry

HFS of the VTA did not significantly alter dopamine levels in the NAc prior to fentanyl administration, a finding that differs from our previous work, wherein HFS of the VTA resulted in significant reductions of tonic dopamine levels in the NAc, even prior to any drug exposure^24^. It should be noted that the VTA is a highly heterogeneous brain structure in both rats and humans, comprising lateral and medial regions with a majority of combinatorial dopaminergic-glutamatergic neurons concentrated near midline^37–39^. Approximately 60–65% of the VTA consists of dopaminergic, 35% GABA, and 3% glutamatergic neurons^39^. Given this cellular diversity, it is possible that differences in electrode placement between studies may have led to selective stimulation of distinct neuronal populations, contributing to the observed variations in NAc dopamine responses. This study utilized a concentric stimulating electrode, providing a smaller more precise stimulation area. Prior work used a forked bipolar stimulating electrode with a broader area of activation, potentially inhibiting a larger proportion of neuronal populations within the VTA by a mechanism involving depolarization block. Our results suggest that VTA HFS may be inhibiting distinct dopaminergic and GABAergic neuronal populations within the VTA^7^. Inhibition of GABAergic neurons is expected to increase dopamine release in the NAc, whereas inhibition of dopaminergic populations would likely decrease dopamine levels in the NAc^6^,^7^.

Future research should explore a broader range of stimulation parameters, including variations in pulse width and amplitude, to improve translational relevance for human applications. Additionally, pharmacological validation may help clarify the mechanisms underlying VTA HFS effects on dopamine levels in the NAc.

### HFS of VTA prevents respiratory failure in response to fentanyl

HFS of the VTA failed to prevent fentanyl-induced respiratory depression, as previously observed with oxycodone^24^. However, this result is not entirely unexpected given the potency of fentanyl, approximately 50 times that of oxycodone^5^. However, importantly we found that HFS of the VTA rescued fentanyl-induced respiratory failure. After a brief period, the respiratory rate climbed up to near baseline and remained stable after stimulation ceased, see (Fig. 4C, 4D). Without HFS, all rats administered 30 μg/kg of fentanyl experienced significant respiratory depression that did not recover see (Fig. 4A, 4B).

The underlying mechanism of this respiratory rescue remains unclear. We hypothesize that due to the intensity of the current applied to the VTA stimulating electrode, the nearby substantia nigra (SN) may also be affected^40^. The SN has known anatomical projections to respiratory control centers, including the pre-Bötzinger complex(preBötC)^41^, the interneurons of which have direct connections to caudal bulbospinal neurons that generate rhythm to the premotor neurons responsible for transmitting oscillatory drive to spinal respiratory motoneurons^42^,^43^. The preBötC contains MORs, when activated by fentanyl, neuronal activity is inhibited and this can lead to acute respiratory failure at high doses^44^. Previous work demonstrated that HFS of the VTA completely attenuates oxycodone-induced respiratory depression^24^. However, in response to a lethal dose of fentanyl, HFS of the VTA still resulted in significant fentanyl-induced respiratory depression but was able to recover. This outcome is not entirely unexpected, given fentanyl’s extreme potency and distinct mechanism of action compared to oxycodone^5,^. Future research should investigate whether selective optogenetic modulation of distinct neuronal cell bodies in the VTA or axons from the medial forebrain bundle (a region distal of both the VTA and SN complex) are able to prevent fentanyl induced respiratory depression alone or if the observed effects are mediated by activation of SN projections to the preBötC.

### Study Limitations

This preclinical study was conducted in anesthetized, drug-naïve male rats rather than a chronic addiction model, limiting our ability to assess the clinical relevance of VTA stimulation in a drug-self-administering animal model. While an acute high dose of fentanyl was purposely applied to emulate an overdose condition in both neurochemical and respiratory response, this approach limited the ability to determine the actual impact of fentanyl on dopamine levels in the NAc compared to respiratory depression induced hypoxia in the brain. Additionally, experiments were completed in urethane anesthetized rats which as previously mentioned in the methods have a significant impact on respiration^32,46,47,48^. As a result, we were limited to using a relatively low dose of fentanyl compared to previous studies on awake rats^49^. Furthermore, previous research has demonstrated significant sex differences in drug-response^49^,^50^, which were not accounted for in this study.

## Conclusions

There are three main conclusions for this study. First, fentanyl at the selected dose and under the present experimental conditions results in significant respiratory depression that, in turn, results in 100% mortality. In addition, HFS of the VTA does not significantly alter tonic and fentanyl-induced increases in NAc dopamine levels but VTA HFS can fully rescue fentanyl-induced respiratory failure. Furthermore, the novel voltammetric technique M-CSWV, in combination with CFMs, provides a reliable approach for measuring extracellular dopamine dynamics with high spatiotemporal resolution. Overall, these provide early preliminary evidence for the potential of DBS as a therapeutic strategy for TROUD and fentanyl-induced respiratory depression.
